# 1-Chloro-1*H*-1,2,3-benzotriazole

**DOI:** 10.1107/S1600536812044820

**Published:** 2012-11-24

**Authors:** Ming-Yong Yuan, Xia Zhao, Ling-Li Zheng

**Affiliations:** a Department of Pharmacy, The First Affiliated Hospital, Chengdu Medical College, Chengdu 610500, People’s Republic of China

## Abstract

The title compound, C_6_H_4_ClN_3_, is essentially planar, with a maximum deviation of 0.007 (3) Å. In the crystal, a short contact of 2.818 (3) Å is observed between N and Cl atoms of adjacent mol­ecules.

## Related literature

For related structures of benzotriazole derivatives, see: Jebas *et al.* (2012 ▶); Guo *et al.* (2012 ▶); Selvarathy *et al.* (2012 ▶); Xu & Shen (2012 ▶). For applications of the title compound, see: Hunter *et al.* (2006 ▶) and references cited therein. For the biological activity of benzotriazole derivatives, see: Gaikwad *et al.* (2012 ▶); Dubey *et al.* (2011 ▶).
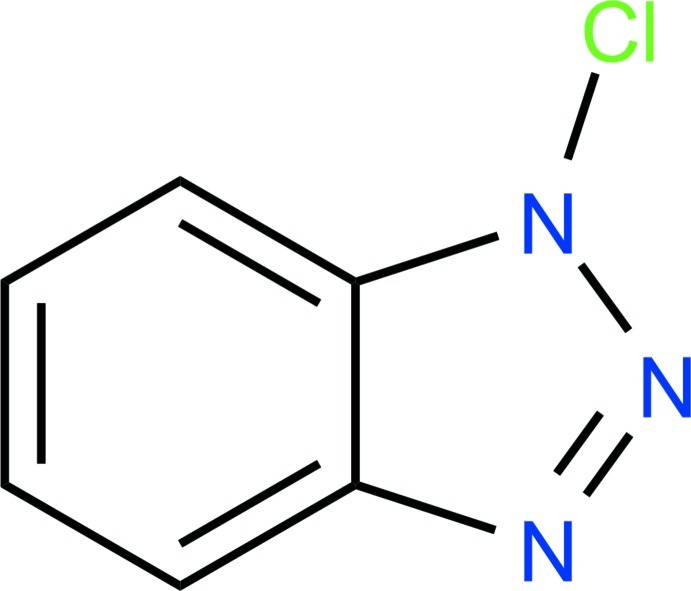


## Experimental

### 

#### Crystal data

C_6_H_4_ClN_3_
*M* *_r_* = 153.57
Orthorhombic, 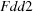

*a* = 22.8022 (11) Å
*b* = 14.2637 (8) Å
*c* = 8.2259 (4) Å
*V* = 2675.4 (2) Å^3^
*Z* = 16
Mo *K*α radiation
μ = 0.48 mm^−1^
*T* = 293 K
0.42 × 0.34 × 0.32 mm

#### Data collection

Agilent Xcalibur Eos diffractometer
Absorption correction: multi-scan (*CrysAlis PRO*; Agilent, 2010 ▶) *T*_min_ = 0.979, *T*_max_ = 1.000
1503 measured reflections
918 independent reflections
867 reflections with *I* > 2σ(*I*)
*R*_int_ = 0.017

#### Refinement

*R*[*F* ^2^ > 2σ(*F* ^2^)] = 0.030
*wR*(*F* ^2^) = 0.060
*S* = 1.06
918 reflections
91 parameters
1 restraint
H-atom parameters constrained
Δρ_max_ = 0.15 e Å^−3^
Δρ_min_ = −0.16 e Å^−3^
Absolute structure: Flack (1983 ▶), 275 Friedel pairs
Flack parameter: 0.00 (8)

### 

Data collection: *CrysAlis PRO* (Agilent, 2010 ▶); cell refinement: *CrysAlis PRO*; data reduction: *CrysAlis PRO*; program(s) used to solve structure: *SHELXS97* (Sheldrick, 2008 ▶); program(s) used to refine structure: *SHELXL97* (Sheldrick, 2008 ▶); molecular graphics: *OLEX2* (Dolomanov *et al.*, 2009 ▶); software used to prepare material for publication: *publCIF* (Westrip, 2010 ▶).

## Supplementary Material

Crystal structure: contains datablock(s) global, I. DOI: 10.1107/S1600536812044820/xu5634sup1.cif

Structure factors: contains datablock(s) I. DOI: 10.1107/S1600536812044820/xu5634Isup2.hkl

Supplementary material file. DOI: 10.1107/S1600536812044820/xu5634Isup3.cml

Additional supplementary materials:  crystallographic information; 3D view; checkCIF report

## Figures and Tables

**Fig. 1.**
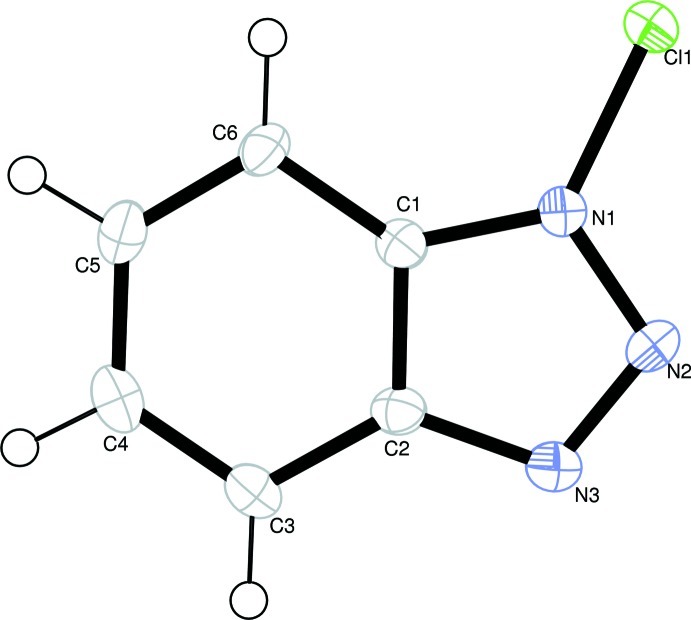
The title compound with displacement ellipsoids drawn at the 50% probability level.

**Fig. 2.**
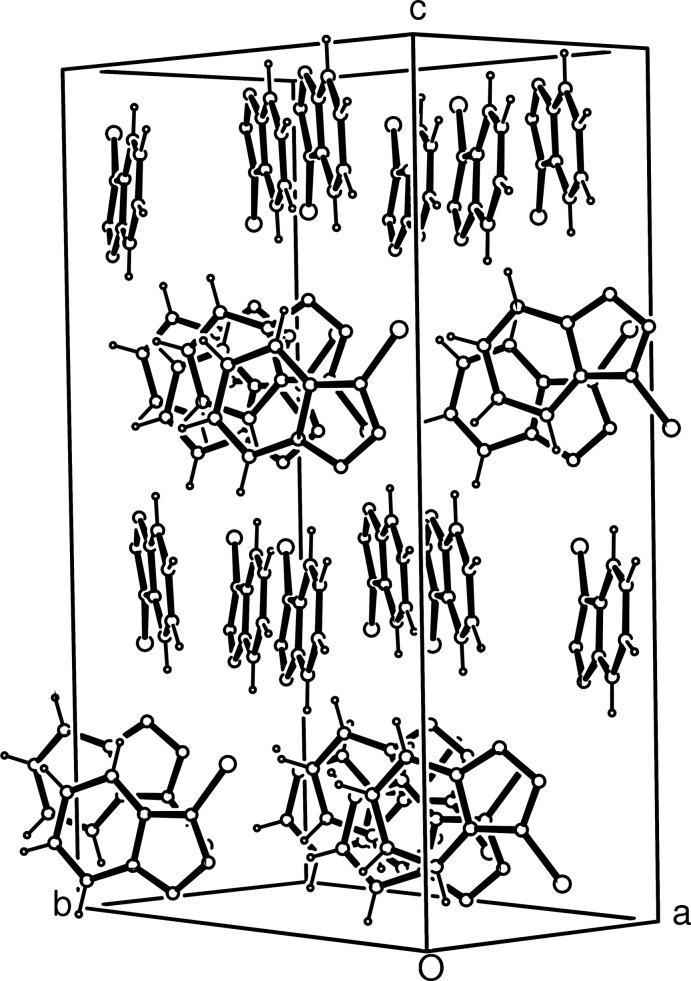
Plane-to-plane stacking of alternate molecules parallel to the α axis.

## supplementary crystallographic information

### Comment

Benzotriazole derivates are an important class of heterocylic compounds with essential applications in the organic synthesis and medicinal chemistry. In the synthetic chemistry, 1-chloro-1*H*-benzo[*d*][1,2,3]triazole is an important oxidation and chlorination reagent. Recently, 1-chloro-1*H*-benzo[*d*][1,2,3]triazole has been used in the synthesis of unsymmetrical disulfides (Hunter *et al.*, 2006). Meanwhile, benzotriazole derivates derivates exhibit numerous essential bioactivitities, especially in antimicrobial (Gaikwad, *et al.*, 2012) and antibubercular activities (Dubey *et al.* 2011). Most recently, several crystal structures of title compound derivates have been reported (Jebas *et al.*, 2012; Guo *et al.*, 2012; Selvarathy *et al.*, 2012; Xu & Shen 2012), but crystal data of 1-chloro-1*H*-benzo[*d*][1,2,3]triazole has not been investigated. Herein, we report the synthesis and crystal structure of the title compound.

The molecular structure of 1-chloro-1*H*-benzo[*d*][1,2,3]triazole is shown in Fig. 1. The bond lengths and angles are within normal ranges. In the crystal, the short contact of 2.818 (3) Å between N and Cl atoms of adjacent molecules occurs.

### Experimental

To a stirring solution of benzotriazole (10 g) in 50 ml of 50% acetic acid aqueous solution was added sodium hypochlorite solution (30 ml) at room temperature dropwise. After dropping, the solution was diluted with water (100 ml) to precipitate the product. The mixture was filtered, washed with water to afford 1-chloro-1*H*-benzo[*d*][1,2,3]triazole (8.5 g)as white solid. The single crystals of 1-chloro-1*H*-benzo[*d*][1,2,3]triazole were recrystallized from acetone at room temperature.

### Refinement

H atoms were included in idealized positions and refined using a riding-model approximation, with C—H = 0.93 Å and *U_iso_*(H) = 1.2*U*_eq_(C).

### Crystal data

**Table:**

C_6_H_4_ClN_3_	*D*_x_ = 1.525 Mg m^−^^3^
*M**_r_* = 153.57	Mo *K*α radiation, λ = 0.71073 Å
Orthorhombic, *F**d**d*2	Cell parameters from 738 reflections
*a* = 22.8022 (11) Å	θ = 3.0–28.5°
*b* = 14.2637 (8) Å	µ = 0.48 mm^−^^1^
*c* = 8.2259 (4) Å	*T* = 293 K
*V* = 2675.4 (2) Å^3^	Block, colourless
*Z* = 16	0.42 × 0.34 × 0.32 mm
*F*(000) = 1248	

### Data collection

**Table:**

Agilent Xcalibur Eos diffractometer	918 independent reflections
Radiation source: fine-focus sealed tube	867 reflections with *I* > 2σ(*I*)
Graphite monochromator	*R*_int_ = 0.017
Detector resolution: 16.0874 pixels mm^-1^	θ_max_ = 25.2°, θ_min_ = 3.0°
ω scans	*h* = −15→27
Absorption correction: multi-scan (*CrysAlis PRO*; Agilent, 2010)	*k* = −16→8
*T*_min_ = 0.979, *T*_max_ = 1.000	*l* = −9→9
1503 measured reflections	

### Refinement

**Table:**

Refinement on *F*^2^	Secondary atom site location: difference Fourier map
Least-squares matrix: full	Hydrogen site location: inferred from neighbouring sites
*R*[*F*^2^ > 2σ(*F*^2^)] = 0.030	H-atom parameters constrained
*wR*(*F*^2^) = 0.060	*w* = 1/[σ^2^(*F*_o_^2^) + (0.0242*P*)^2^] where *P* = (*F*_o_^2^ + 2*F*_c_^2^)/3
*S* = 1.06	(Δ/σ)_max_ = 0.001
918 reflections	Δρ_max_ = 0.15 e Å^−^^3^
91 parameters	Δρ_min_ = −0.16 e Å^−^^3^
1 restraint	Absolute structure: Flack (1983), 275 Friedel pairs
Primary atom site location: structure-invariant direct methods	Flack parameter: 0.00 (8)

### Special details

**Table:**

Geometry. All esds (except the esd in the dihedral angle between two l.s. planes) are estimated using the full covariance matrix. The cell esds are taken into account individually in the estimation of esds in distances, angles and torsion angles; correlations between esds in cell parameters are only used when they are defined by crystal symmetry. An approximate (isotropic) treatment of cell esds is used for estimating esds involving l.s. planes.
Refinement. Refinement of F^2^ against ALL reflections. The weighted R-factor wR and goodness of fit S are based on F^2^, conventional R-factors R are based on F, with F set to zero for negative F^2^. The threshold expression of F^2^ > 2sigma(F^2^) is used only for calculating R-factors(gt) etc. and is not relevant to the choice of reflections for refinement. R-factors based on F^2^ are statistically about twice as large as those based on F, and R- factors based on ALL data will be even larger.

### Fractional atomic coordinates and isotropic or equivalent isotropic displacement parameters (Å^2^)

**Table:**

	*x*	*y*	*z*	*U*_iso_*/*U*_eq_	
Cl	0.31785 (3)	0.13250 (5)	−0.13509 (9)	0.0508 (2)	
N1	0.37734 (9)	0.18891 (16)	−0.0618 (3)	0.0436 (6)	
N2	0.43133 (10)	0.15132 (19)	−0.0788 (3)	0.0544 (7)	
N3	0.46849 (10)	0.20933 (18)	−0.0121 (4)	0.0537 (7)	
C1	0.37873 (11)	0.27246 (19)	0.0179 (3)	0.0369 (6)	
C2	0.43801 (11)	0.2849 (2)	0.0484 (3)	0.0402 (7)	
C3	0.45739 (14)	0.3651 (2)	0.1311 (4)	0.0538 (8)	
H3	0.4969	0.3751	0.1531	0.065*	
C4	0.41526 (15)	0.4282 (2)	0.1780 (4)	0.0577 (9)	
H4	0.4265	0.4822	0.2330	0.069*	
C5	0.35617 (14)	0.4133 (2)	0.1453 (4)	0.0550 (8)	
H5	0.3292	0.4581	0.1796	0.066*	
C6	0.33580 (13)	0.3357 (2)	0.0649 (4)	0.0459 (8)	
H6	0.2962	0.3262	0.0435	0.055*	

### Atomic displacement parameters (Å^2^)

**Table:**

	*U*^11^	*U*^22^	*U*^33^	*U*^12^	*U*^13^	*U*^23^
Cl	0.0413 (4)	0.0522 (4)	0.0587 (4)	−0.0073 (4)	−0.0014 (4)	−0.0109 (4)
N1	0.0307 (12)	0.0452 (15)	0.0549 (15)	−0.0005 (12)	−0.0016 (12)	−0.0116 (13)
N2	0.0396 (15)	0.0519 (17)	0.072 (2)	0.0078 (12)	0.0062 (13)	−0.0114 (15)
N3	0.0327 (12)	0.0566 (16)	0.0719 (16)	0.0032 (14)	−0.0019 (13)	−0.0092 (13)
C1	0.0367 (15)	0.0383 (16)	0.0358 (14)	0.0036 (14)	−0.0016 (12)	0.0000 (13)
C2	0.0368 (15)	0.0404 (16)	0.0434 (14)	−0.0002 (13)	−0.0010 (14)	0.0022 (13)
C3	0.0494 (18)	0.057 (2)	0.0545 (19)	−0.0129 (16)	−0.010 (2)	−0.0014 (17)
C4	0.079 (2)	0.0376 (18)	0.0566 (19)	−0.0060 (18)	−0.002 (2)	−0.0069 (15)
C5	0.0570 (19)	0.0439 (19)	0.064 (2)	0.0099 (16)	0.0071 (18)	−0.0067 (18)
C6	0.0385 (15)	0.0440 (19)	0.0551 (18)	0.0071 (14)	−0.0006 (14)	−0.0004 (16)

### Geometric parameters (Å, º)

**Table:**

Cl—N1	1.688 (2)	C3—C4	1.371 (4)
N1—N2	1.350 (3)	C3—H3	0.9300
N1—C1	1.360 (3)	C4—C5	1.390 (4)
N2—N3	1.305 (3)	C4—H4	0.9300
N3—C2	1.376 (4)	C5—C6	1.371 (4)
C1—C2	1.386 (3)	C5—H5	0.9300
C1—C6	1.386 (4)	C6—H6	0.9300
C2—C3	1.403 (4)		
			
N2—N1—C1	112.1 (2)	C4—C3—H3	121.6
N2—N1—Cl	120.4 (2)	C2—C3—H3	121.6
C1—N1—Cl	127.46 (18)	C3—C4—C5	121.6 (3)
N3—N2—N1	107.3 (2)	C3—C4—H4	119.2
N2—N3—C2	108.7 (2)	C5—C4—H4	119.2
N1—C1—C2	102.8 (2)	C6—C5—C4	123.0 (3)
N1—C1—C6	133.5 (3)	C6—C5—H5	118.5
C2—C1—C6	123.7 (3)	C4—C5—H5	118.5
N3—C2—C1	109.1 (3)	C5—C6—C1	114.9 (3)
N3—C2—C3	131.0 (3)	C5—C6—H6	122.5
C1—C2—C3	120.0 (3)	C1—C6—H6	122.5
C4—C3—C2	116.8 (3)		
